# Inter-pregnancy interval after caesarean section and subsequent pregnancy outcomes in New South Wales. A data linkage cohort protocol

**DOI:** 10.1016/j.mex.2026.103887

**Published:** 2026-03-27

**Authors:** Alison M Canty, Annemarie Hennessy AM, Wenpeng You, Hazel Keedle, Angela Makris

**Affiliations:** a Western Sydney University, School of Medicine, Penrith, NSW, Australia; b Western Sydney University School of Nursing and Midwifery, Penrith, NSW, Australia; c University of Sydney, Faculty of Medicine and Health, Sydney, NSW, Australia; d University of Adelaide, School of Nursing, Adelaide, SA, Australia; e University of Adelaide, School of Medicine, Adelaide, SA, Australia

**Keywords:** Population study, Interpregnancy interval, Caesarean, Pregnancy outcome, Data linkage, Interbirth interval, Cohort

## Abstract

Caesarean section (CS) rates are increasing worldwide. CS and repeat caesarean alter the risk profile for next pregnancy. Interpregnancy interval (IPI) has been shown to modify maternal and neonatal risk in subsequent pregnancies. There is limited research in the Australian context addressing outcomes relative to interpregnancy interval. The lack of pregnancy spacing information adds to confusion and conflicting recommendations for maternity care providers and women. We developed a protocol using data linkage of routinely collected hospital data from 1994 to 2023 in New South Wales, Australia to investigate both maternal and neonatal outcomes relative to interpregnancy intervals.

• Large scale study of interpregnancy interval after CS in an Australian multiethnic cohort not previously explored
• Quantify the risks associated with differing interpregnancy intervals compared to contemporaneous controls in this cohort
• Results presented as absolute risk facilitating access to evidence-based information

## Graphical abstract

**Figure:**
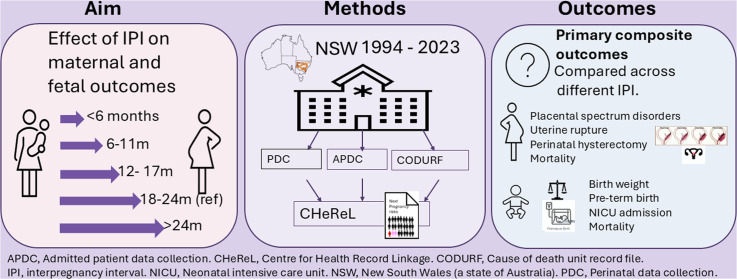

**Table:** Specifications table.

**Subject area**	Medicine and Dentistry
**More specific subject area**	Women’s health
**Name of your protocol**	Inter-pregnancy interval after caesarean section, incidence, and outcomes in New South Wales (NSW).
**Reagents/tools**	The Centre for Health Record Linkage (CHeReL) New South Wales Ministry of Health, for access to linked data https://www.cherel.org.au/ Secure Unified Research Environment (SURE) supplied through the SAX institute.
**Experimental design**	Retrospective cohort using routinely collected population data.
**Trial registration**	NA
**Ethics**	Ethics approval is dated from the 4th of February 2025, from the NSW Population and Health Services Research Ethics Committee 2024/ETH00864 / 2024.16. As this project contains waiver of consent publication and plain language summaries will be disseminated.
**Value of the Protocol**	Provides the first large-scale Australian, multiethnic population-based assessment of interpregnancy interval following caesarean section Quantifies maternal and neonatal risks using absolute risk measures to support clinical counselling and shared decision-making Demonstrates the use of linked administrative data to inform pregnancy-spacing policy and co-designed consumer resources

## Background

Caesarean section (CS) rates are increasing worldwide [1]. The World Health Organization recommendation is for a CS rate of up to 15% [2]. This is exceeded in Australia where the CS rate has risen from 32.3% in 2011 to 41% in 2023 [3]. The most common reason for repeat CS in Australia is previous caesarean [3]. Repeat CS increases the risk of placenta previa and placenta accreta spectrum (PAS) disorders including accreta, increta and percreta [4,5]. Following caesarean section, uterine scar healing introduces a biologically plausible mechanism through which short interpregnancy intervals may confer additional maternal and neonatal risk. Furthermore, both PAS disorders and uterine rupture contribute to peripartum or caesarean hysterectomy [[5], [6]].

There is limited research in the Australian context addressing outcomes relative to interpregnancy interval (IPI). Internationally several outcomes associated with IPI, maternal and / or neonatal have been investigated, with variation in findings. Maternal morbidity and mortality was found to have an increased risk aRR 1.18 (95%CI, 1.09–1.27) with an IPI of 6 months as compared to 18 months after all modes of birth in a population study in Canada [7]. Longer IPI has been shown to decrease the risk of uterine rupture [8] and increase successful vaginal birth after caesarean (VBAC) [9,10].

Shorter IPI of approximately 6 months or less, after all modes of birth, has been found to have implications for neonates including pre-term birth, small for gestational age and increased perinatal mortality [7,11,12]. A 2023 population-based study across 46 countries found perinatal mortality (from 28 weeks gestation to 7 days after birth) after an IPI of <6 months had an all-inclusive hazard ratio of 2.72 (95% CI 2.52–2.93) compared to 18 to 23 months, with no significant difference in perinatal mortality between an IPI of 18 to 23 months and 24 to 59 months [12].

Ideal recommended IPI and potential risks posed by short IPI have not been well identified by women [[13], [14], [15]]. Additionally, sharing of information by maternity care providers on risk and benefit for next birth after caesarean (NBAC) has been reported as lacking [15,16]. The aim of this study is to determine the effect of different IPI after CS and the effect on maternal and neonatal outcomes of the next pregnancy across New South Wales (NSW) and to compare these outcomes with published data from similar health systems internationally. Absolute risk results from this data linkage project will be made available to a co-design project including women with lived experience and health care professionals providing maternity care undertaking a project to develop an accessible evidence-based IPI resource.

## Description of protocol

This study is designed using routinely collected health data across NSW, Australia. The study setting includes secondary routinely collected hospital data across one state, NSW, via the Centre for Health Research Linkage (CHeReL) database data linkage across the NSW perinatal data collection (PDC), NSW admitted patient data (APDC) and cause of death unit record file (CODURF) data bases, Table 1. Access to these databases is via application to both the CHeReL and the NSW Population and Health Services Research Ethics Committee. Greater detail on available data within these data sets is available at https://www.cherel.org.au/datasets.

**Table 1:** Description of data sets included in data linkage.

Dataset	Description	Data custodian
New South Wales Perinatal Data Collection (NSWPDC)	Birth data collected from all homebirths, public and private hospitals across NSW of all live and still births of at least 20 weeks gestation or 400gm birth weight. Data requested include mode of birth, discharge status, maternity service level.	Centre for Health Record Linkage (CHeReL)
New South Wales Admitted Patient Data Collection (NSWAPDC)	A record of all discharges from public and private hospitals, multi-purpose services and private day procedure centers across NSW. Data includes but not limited to, length of stay, admission to ICU, diagnosis codes using ICD-10_AM, and demographic information.	Centre for Health Record Linkage (CHeReL)
Cause of Death Unit Record File (CODURF)	All deaths recorded within NSW. Data requested includes underlying cause of death, contributing causes of death.	Centre for Health Record Linkage (CHeReL)

The cohort for this study includes women who have had a primary CS or VBAC between January 2014 to latest available in 2023 the study population will then include (Supplementary file):

• Women with a prior a CS, including those with subsequent pregnancies resulting in abortive outcomes, or liveborn or stillborn infants at ≥20 weeks’ gestation, and their outcomes.

The outcome of interest is time to next pregnancy, the reference group being 18 to 23 months. IPI will be categorized into <6, 6 to11, 12 to17, 18 to 23, 24 to 59 and 60 months or more. Secondary outcomes include comparisons between IPI categories across a range of maternal and neonatal outcomes. Maternal outcomes included uterine rupture, placental spectrum disorders, perinatal hysterectomy and mortality. Neonatal outcomes include birth weight for gestational age, pre-term infant, neonate requiring higher level care after birth and mortality. Stratification will occur by including, but not limited to, maternal age, socio-economic area of residence, body mass index (BMI) and parity.

The expected number of women to be included will be up to 157,426. The sample size has been estimated based on annual incidence of multiparous women with a history of CS birthing in NSW 2014 to 2023 [3]. Previous studies have reported IPI of <6months in 3.7 to 11.7% of their study population [7,[17], [18], [19]], Conde-Agudelo (2000) reports an incidence of IPI <6months as 11.7% amongst women with previous CS [18]. The cohort of multiparous pregnancies with a history of CS over the 10 years will be approximately 157,426 and pregnancies with a short IPI will make up approximately 11,019 pregnancies. There is wide variation in incidence of uterine rupture relative to spontaneous onset of labour and differing induction methods, from 0.19% – 1.91% [20,21]. Based on a 0.35% (average) rate of uterine rupture in the control population, we will be able to detect a true odds ratio of between 0.551 - 1.528 in women with short IPI relative to controls with a probability of 0.8 assuming a Type I error of 0.05. The study will be adequately powered to test whether 0.5% remains a valid reflection of uterine rupture as per NSW Health NBAC guidance given changes in population demographics and approaches to repeat CS over time. We will use a continuity-corrected chi-squared statistic to evaluate the null hypothesis. Therefore, we expect to have sufficient power to address the aim of this study.

Using a between-subject design, data will be analyzed using descriptive statistics on SPSS© software accessed securely via the SAX Institute Secure Unified Research Environment (SURE). Descriptive data will be described using either means or standard deviations, median, interquartile range or count dependent on data type and distribution. IPI categories will be compared to the reference group 18–23 months. IPI groups that will be compared include <6 months, 6 to 11 months, 12 to 17 months, 24 to 59 months and 60 months or more. IPI will be defined as from the previous live or still birth to conception of the next pregnancy. For women within the cohort who have three pregnancies identified, a within-subject statistical model can also be undertaken in relation to differences in birth outcomes including gestational age at birth or small for gestational age, in line with recommendations addressing confounding, accounting for both ethnicity and genetic factors that persist with a woman throughout her reproductive life [17,22]. Statistical significance of outcomes will include clinical and public health significance as suggested by Hutcheon [22] adding relevance to policy and public health approaches.

Missing data of past gynecological history e.g. any uterine or cervical surgery will not necessarily be available via the population data sets examined. Therefore, changes in gravidity relative to parity at the next pregnancy will be used as a proxy for pregnancy loss. Pregnancy loss in this time frame may lead to a longer IPI or altered risk profile, requiring sensitivity analysis. Considerations for addressing potential bias, effect modifiers or confounding and controlling for between-subject design include, but are not limited to age, socio economic area, BMI, smoking history and number of previous CS. Adjustment for confounding of ordinal data will be undertaken using the mantel-Haenszel chi-square test and regression such as logistic regression. Regression analyses will be used to estimate adjusted associations, while stratified analyses will be used to explore potential effect modification. Using IPI (birth to conception) as opposed to interbirth interval (IBI) (birth to birth) to reduce the bias created by short IBI relative to preterm birth. Presentation of findings of IPI as a categorical variable will assist in comparison with previous IPI studies. IPI will be calculated from last birth to next pregnancy with a recorded last menstrual period (LMP) [23]. If LMP is not available this will be calculated from the expected date of confinement from that pregnancy. Analyzed data will be presented and reported as absolute risk between IPI categories (risk difference number of cases per 100) [22] presenting a clinical or public health approach to potential difference in risk.

Where missing data comprises <10% of total dataset, complete case analysis (listwise deletion) will be undertaken under the assumption that data are missing at random (MAR)***.*** This assumption will be assessed descriptively where possible.

Consumers were not involved in the design of this protocol; it is intended that the results of this data linkage project will be available to a co-design project including women with lived experience and health care professionals providing maternity care. The analyzed data will add to the knowledge base of IPI after CS for clinicians, women and their families regarding planning a next pregnancy after a CS and can be used to inform policy around pregnancy planning after CS.

### Protocol validation

This data linkage project aims to bring together data from the outcomes of subsequent pregnancies including both maternal and neonatal outcomes relative to next pregnancy after a CS in the current birthing climate within Australia. Data linkage has been shown to be a useful resource for the purpose of comparable birth outcomes [23], and hospital coded data is reliable for conditions arising in pregnancy birth [24]. This will provide a picture of one aspect of maternity care in NSW and the potential impacts on women, their families and the health system in coping with the continued rise in the incidence and prevalence of CS and its associated outcomes.

## Limitations

• Exclusion of women who have vaginal birth and an unscarred uterus
• Possibility of missing data (e.g. Births that occurred outside of New South Wales within the study period) or errors within linked data, history of gynecological surgery and pregnancy loss

## CRediT author statement

**Alison Canty**: Conceptualization, methodology, writing original draft, writing-review & editing, project administration. **Annemarie Hennessy:** Conceptualization, methodology, writing-review & editing, supervision. **Hazel Keedle:** Conceptualization, methodology writing-review & editing, supervision. **Wenpeng You**: methodology, writing-review & editing. **Angela Makris:** Conceptualization, methodology, writing-review & editing, project administration, supervision.

## Declaration of interests

The authors declare that they have no known competing financial interests or personal relationships that could have appeared to influence the work reported in this paper.

## Data Availability

The authors do not have permission to share data.

## Appendix Supplementary materials

Supplementary material *and/or* additional information [OPTIONAL] Image, application 1
