# Supplementary material for: Inter-pregnancy interval after caesarean section and subsequent pregnancy outcomes in New South Wales. A data linkage cohort protocol
Source: MethodsX. 2026 Mar 27;16:103887. doi: 10.1016/j.mex.2026.103887 (PMC13068596; doi:10.1016/j.mex.2026.103887)
Supplement: Supplementary file 1 — Supplementary material and/or additional information [OPTIONAL] [file mmc1.docx]

**Supplementary table 1**

**Data linkage cohort definition, linkage variables and outcome variables.**

| **Data linkage cohort definition summary** |
| --- |
| ***NSW Perinatal Data Collection*** |
| **IPIPLINKAGE Cohort (2014 to 2023)** |
| Was last birth by caesarean section? |
| Type of delivery, caesarean section |
| **Linkage variables summary** |
| **1994 to latest available** |
| ***Australian Coordinating Registry Cause of Death Unit Record File (NSW registered deaths)*** |
| Date of death |
| Age at death |
| Place of birth |
| Period of residence in Australia |
| Sex |
| ***NSW Perinatal Data Collection*** |
| Mother's date of birth |
| Mother's country of birth |
| Mother's marital status |
| Mother's Statistical Area of residence |
| Mother's Local Health District of residence |
| Local Health District of Hospital |
| Baby's date of birth |
| Expected date of confinement |
| Previous pregnancy |
| Number of previous pregnancies |
| Was last birth by caesarean section? |
| Total number of previous caesarean section |
| Antenatal care |
| Duration of pregnancy at first antenatal visit |
| Number of antenatal visits |
| Smoking |
| Mother's height |
| Mother's weight |
| Maternal diabetes |
| Maternal hypertension |
| Post-partum haemorrhage |
| Blood transfusion flag |
| Estimated blood loss |
| Baby length of stay |
| Labour onset |
| Induction and augmentation of labour |
| Analgesia for labour |
| Anaesthesia for delivery |
| Type of delivery |
| Main indication for caesarean section |
| Perineal status |
| Main model of care |
| Baby's sex |
| Plurality of birth |
| Birth order |
| Gestational age |
| Birthweight |
| APGAR score (1 min) |
| APGAR score (5 min) |
| Resuscitation of baby |
| Baby's discharge status |
| Perinatal death type |
| Baby's place of birth |
| Mother's discharge status |
| Confinement based on first baby |
| Baby feeding on discharge |
| ***NSW Admitted Patient Data Collection*** |
| Hospital type |
| Facility type |
| Peer group |
| Local Health District of Facility |
| Facility identifier |
| Stay number (encrypted) |
| Episode sequence number |
| Episode length of stay |
| Episode of care type |
| Mode of separation |
| DRG mode of separation |
| Facility transferred to |
| Emergency status |
| Emergency Department Status |
| Source of referral |
| Unit type on admission |
| Hours in ICU |
| Hours on mechanical ventilation |
| Involuntary days in psychiatric unit |
| Last psychiatric admission date |
| Days in psychiatric Unit |
| Qualified bed days |
| Australian Refined Diagnosis Related Group |
| AR DRG Version |
| Major Diagnostic Category |
| Service Related Group |
| SRG Version |
| Clinical code set |
| Condition onset flag |
| Diagnosis codes |
| Procedure codes |
| Health insurance on admission |
| Sex |
| Birth date |
| Marital status |
| **Outcome variables summary** |
| **1994 to latest available** |
| ***maternal*** |
| Placenta praevia |
| Antepartum haemorrhage |
| Preterm labour and delivery |
| Induction and augmentation of labour |
| Postpartum haemorrhage |
| Retained placenta |
| Vaginal delivery |
| Forceps and Vacuum |
| Caesarean section |
| Plurality |
| Puerperal infections |
| Date of death |
| Underlying Cause of Death Diagnosis Code |
| Contributing causes of death (ICD-10) |
| ***neonatal*** |
| Gestational age |
| Birth weight |
| Newborns/Neonates with conditions originating in perinatal period |
| Date of birth |
| Date of death |
| Underlying Cause of Death Diagnosis Code |
| Contributing causes of death (ICD-10) |
